# Effect of a carbohydrate lollipop on the gastric volume of fasted pediatric patients

**DOI:** 10.1111/pan.14479

**Published:** 2022-05-20

**Authors:** Pieter Odendaal, Annemie Burke, Johan Coetzee

**Affiliations:** ^1^ Department of Anesthesiology and Critical Care, Tygerberg Academic Hospital University of Stellenbosch Parow South Africa

**Keywords:** carbohydrates, child, fasting, measurement, ultrasound

## Abstract

**Background:**

Preoperative fasting is part of routine practice. Children subjected to prolonged preoperative fasting often suffer adverse effects. Consuming a preoperative lollipop may lessen their anxiety and have clinical benefits.

**Aims:**

To assess the effect of consuming a lollipop on gastric volume and the feasibility of administering a lollipop to a child preoperatively.

**Methods:**

In this prospective, repeated measures interventional study, we measured gastric antrum volume using ultrasound in children aged 2–18 years. We measured antrum volumes after participants had fasted for a minimum of 6 h for solids and 2 h for clear fluids. They then consumed a standard carbohydrate lollipop, and we repeated the antrum volume measurements after 1 h.

**Results:**

Of the 38 patients enrolled, 32 completed the study; four had ingested additional food or liquid, and two were diagnosed with systemic disease the day after data collection. The gastric volume data were normally distributed. The mean volume change was 0.01 ml kg^−1^ (95% CI −0.02 to 0.05; *p* = .460). The mean postlollipop volume was 0.51 ml kg^−1^ (95% CI 0.43 to 0.58).

**Conclusions:**

Consuming a standard lollipop did not affect the gastric volume of fasted pediatric patients.

**What is already known about the topic?**

Routine preoperative fasting is often prolonged in elective pediatric surgery, despite pulmonary aspiration being rare. The influence of a lollipop on gastric volume has not been accurately investigated.

**What new information this study adds?**

Children's gastric volumes do not increase 1 h after consuming a pure carbohydrate lollipop.

## 1 INTRODUCTION

Preoperative fasting is routine practice before surgery and is intended to decrease the risk of aspiration.^1^ Pulmonary aspiration of gastric content may occur through a combination of absent airway reflexes and passive regurgitation of gastric content during anesthesia. It can be catastrophic and lead to hypoxemia, prolonged ventilation, cardiac arrest or adverse airway events like bronchospasm and laryngospasm.^2^ In pediatrics, this remains a rare event with incidences of 2–10 per 10 000 patients.^2^

Prolonged fasting increases thirst and irritability,^3^ and can result in detrimental metabolic effects such as hypoglycemia and ketoacidosis.^4^ A clear fluid fasting policy down to 2 h often results in a mean of 7 h of fasting in practice.^5^ Shortening fasting time to 1 h improves a child's metabolism and hemodynamic tolerance to induction of anesthesia, while decreasing postoperative nausea and opioid use.^2^, ^4^, ^6^

Guidelines by North American and European anesthesiology organizations between 1998 and 2011 endorsed a rule of “6‐4‐2” hours of fasting for solids, breast milk, and clear fluids.^7^, ^8^ A 2016 review of the guidelines classified only two recommendations as level A: (1) Shortened preoperative fasting times and (2) Clear fluid consumption allowed down to 2 h preoperatively.^9^ In 2020, after mounting evidence emerged regarding the low incidence of aspiration, and efficacious introduction of a 1 h preoperative fast for clear fluids, an international consensus statement endorsed unrestricted clear fluid 1 h preoperatively in pediatric and adult patients.^10^ Guidelines by the European Society of Anaesthesiology (ESA) in 2011 recommended continuing surgery if boiled sweets were sucked beforehand.^8^ Current guidelines omit this recommendation, as it was extrapolated from chewing gum findings.^1^, ^2^ The effect of preoperative boiled sweets on gastric volume has not yet been investigated.

Point‐of‐Care Ultrasonography (POCUS) of gastric content is a valuable tool for anesthesiologists when a patient's fasting status is unknown.^2^, ^11^, ^12^ The cross‐sectional area (CSA) of the gastric antrum in pediatric patients is readily identified and correlates well with total gastric residual volume (GRV).^2^, ^13^ Mathematical equations for estimating GRV from the antral CSA in pediatric patients^14^, ^15^ have been utilized to investigate the effect of carbohydrate fluids on GRV and gastric emptying times.^16^, ^17^

We hypothesized that consumption of a standard carbohydrate lollipop would not increase the gastric volume of fasted pediatric patients after 1 h. Our aims were to assess the effect of a lollipop on gastric volume and the feasibility of giving a lollipop to a child preoperatively.

## 2 MATERIALS AND METHODS

The Stellenbosch University Health Research Ethics Committee approved the study (10 October 2020, Reference No. S20/05/117). We registered the study with the South African National Health Research Database (https://nhrd.health.gov.za; Reference no. WC_202010_042). It was a single center prospective cohort study, conducted at Tygerberg Hospital, Western Cape, South Africa. We performed the study according to the ethics of the Declaration of Helsinki. Methodology followed recommendations of the TREND statement for reporting nonrandomized studies.^18^

Our primary outcome was to establish whether in fasted children, there is a difference in gastric residual volume per kilogram bodyweight (ml kg^−1^) 1 h after consuming a lollipop. The secondary outcome was to determine whether there was a change in qualitative sonographic gastric antrum grading.

The legal guardians of children aged 2–18 years, scheduled for elective surgical procedures, provided written, informed consent. Children older than 7 years provided additional assent. They consented a day before data collection, to ensure overnight fasting compliance. We collected our data on the morning of the day prior to surgery, while ensuring that participants' daily feeding schedules were not interrupted. Exclusion criteria were any known medical condition, BMI > 35 kg m^−2^, drugs that altered gastric motility, an overnight fasting time <6 h for solids and 2 h for clear fluids, unwillingness to participate on the day, lollipop consumption longer than 1 h, and a desire to ingest food.

Patients were recruited consecutively over 6 months, depending on the availability of the investigator. On the morning of data collection, the legal guardians confirmed the children's fasting status. Investigator PO performed two ultrasound assessments at the children's bedsides; an initial (fasting) assessment, and a second assessment 1 h after a lollipop had been offered to the child, after confirming that the participant had consumed the lollipop and that he/she had not ingested any other foods or liquids. The lollipop was a readily available, pure carbohydrate lollipop (Amos® Lollipop) of 3.5 ml volume consisting of 8 g Carbohydrate, 0 g Protein, <0.1 g Fat.

Investigator PO performed all the focused ultrasound assessments of the gastric antra. He is certified for gastric ultrasound studies and had performed at least 40 gastric ultrasound examinations in addition to more than 5 years of perioperative and critical care ultrasound experience. He used the Sonosite M‐Turbo ® (FUJIFILM Sonosite, Inc.) with a 6–13 MHz linear transducer.

Each examination was performed with the child placed first in the supine and then in the right lateral decubitus position (RLD). The transducer was placed inferior to the xiphisternum in a sagittal or parasagittal plane, with the probe pointer directed cephalad. The transducer was tilted or rotated to optimize the image of the gastric antrum. The correct plane was identified when the antrum was visualized adjacent to the pancreas or left liver lobe, with the aorta or inferior vena cava visualized in a longitudinal axis.^14^, ^15^

Qualitative grading of the gastric antrum was assessed using the 3‐point grading system described by Perlas et al.^11^ The antrum was described as empty if it appeared flat, with the anterior and posterior walls juxtaposed during a dynamic scan. The antrum was deemed to contain fluid if it appeared to have an endo cavitary lumen with hypoechoic or anechoic content and distended walls. Solid matter was described if echoic content was seen, such as the described “frosted glass” appearance. A grading score of 0, 1, or 2 was applied as follows: Grade‐0—no fluid visible in the antrum in either the supine or RLD position; Grade‐1—antral fluid visualized only in the RLD position.; and Grade‐2—antral fluid visualized in both the supine and RLD position. After the second measurement the participants were allowed to continue with their daily routine.

Quantitative measurements were performed by obtaining three still images of the gastric antrum in the RLD, between peristaltic contractions, as this correlates most strongly with gastric volume.^14^ The cross‐sectional area (CSA) of the antrum, expressed in cm^2^, was measured using the ultrasound machine's internal caliper free‐tracing tool, by tracing the outer layer of the antrum corresponding to the gastric serosa. The mean of the three values obtained from each image was used for gastric volume calculation. We used an equation previously derived by Spencer and colleagues,^14^ to estimate total gastric volume: Gastric volume = −7.8 + (3.5 × RLD CSA in cm^2^) + (0.127) × age in months. Each participant's calculated gastric volume was divided by their weight and expressed as ml kg^−1^.

Data obtained from each participant included age (in months), weight (kg), length (cm), gender, fasting duration (h), if the lollipop had been completely ingested, qualitative grading, presence of solid content and three quantitative CSA values for each still image. One panned video clip of the antrum, the still images in supine and RLD positions, and 3 still images with applicable caliper tracings and CSA in the RLD position were stored electronically for both the fasted and postlollipop ultrasound assessments. We used a combination of qualitative and quantitative assessments to stratify individual risk for aspiration as described by Perlas et al.^11^ A qualitative grading of 0 or 1, with a volume calculation of less than 1.5 ml kg^−1^ constituted a low risk for aspiration. A grade‐2 antrum, or solid matter, or a grade‐1 antrum with a calculated volume of more than 1.5 ml kg^−1^ constituted a high risk for aspiration.

### 2.1 Data analysis

We planned a prospective, repeated measures, interventional study, testing for equivalence. We employed the method of Jones et al.^19^ to calculate the required sample size. For calculation of the expected effect size, we accepted an increase in gastric volume of ≤1.0 ml kg^−1^ (standard deviation 0.84 ml kg^−1^) as constituting low risk for aspiration of gastric contents. We based our 1.0 ml kg^−1^ value firstly on the findings of two studies of fasted children^14^, ^20^ that employed the Spencer et al. equation,^14^ in which mean volume changes were 0.28 and 0.63 ml kg^−1^, and secondly on a gastric volume threshold of 1.5 ml kg^−1^ that is accepted as constituting an increased risk for aspiration.^13^, ^16^. We derived the standard deviation of 0.84 ml kg^−1^ from a study by Song et al.,^16^ who measured antrum volumes in 79 children after ingestion of carbohydrate drinks. For 90% power and two‐sided alpha 0.05, the required sample size is 30 subjects. We intended to recruit 40 participants to allow for dropouts and protocol violations.

We conducted statistical analyses using computer software (MedCalc Statistical Software, version 20.014 (MedCalc Software Ltd; https://www.medcalc.org; 2021)). Table A1 in the Appendix S1 displays the tests employed. We regarded an alpha value of <.05 as indicating statistical significance. We specified a priori that the pre‐ and postlollipop antrum volume measurements would be regarded as equivalent if the 95% confidence interval (95% CI) of the mean volume change was <1.0 ml kg^−1^. Thus, the null hypothesis was that the 95% CI of the mean volume change would be ≥1.0 ml kg^−1^. We also compared pre‐ and postlollipop antrum volumes by conducting a paired *t*‐test, for which the null hypothesis was that there would be no statistically significant difference. We regarded a high risk of aspiration to be present if the postlollipop antrum volume was ≥1.5 ml kg^−1^.

## 3 RESULTS

**Figure 1:**
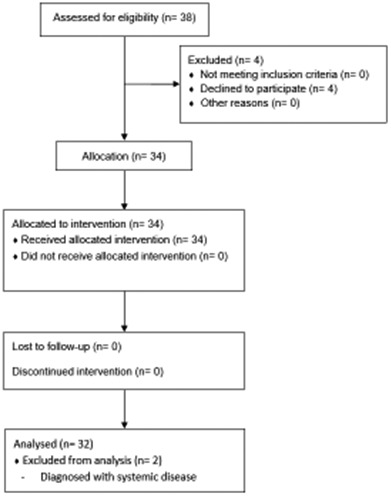
Flow diagram of patient enrolment and analysis.

We recruited 38 participants. The CONSORT flow diagram is shown in Figure 1. Of the six excluded recruits, four opted to eat or drink before the second measurement and two were diagnosed with infectious diseases on the day of their data collection.

A total of 32 ASA physical grading 1 participants were included in the final analysis of whom 23 (72%) were male and 9 (28%) were female. Participant demographics are summarized in Table 1. Procedures the participants underwent are depicted in the Appendix S1 (Table A2).

**Table 1:** Patient demographics

	Mean	SD or IQR	Range
Age (years)	8.3	3.5	2.4–14.3
Weight (kg)	26.3	9.7	11.5–50
Height (cm)	127	20	84–172
Fasting time (h)	10	9–11	6–14

Abbreviations: IQR, interquartile range; SD, standard deviation.

**Figure 2:**
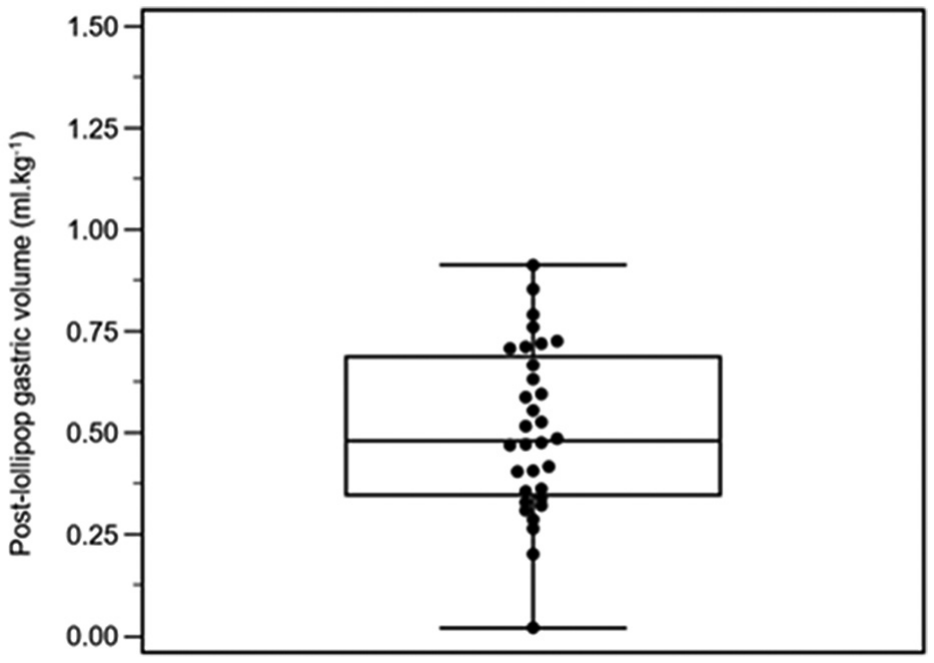
Box and whisker plot of postlollipop gastric volumes.

**Figure 3:**
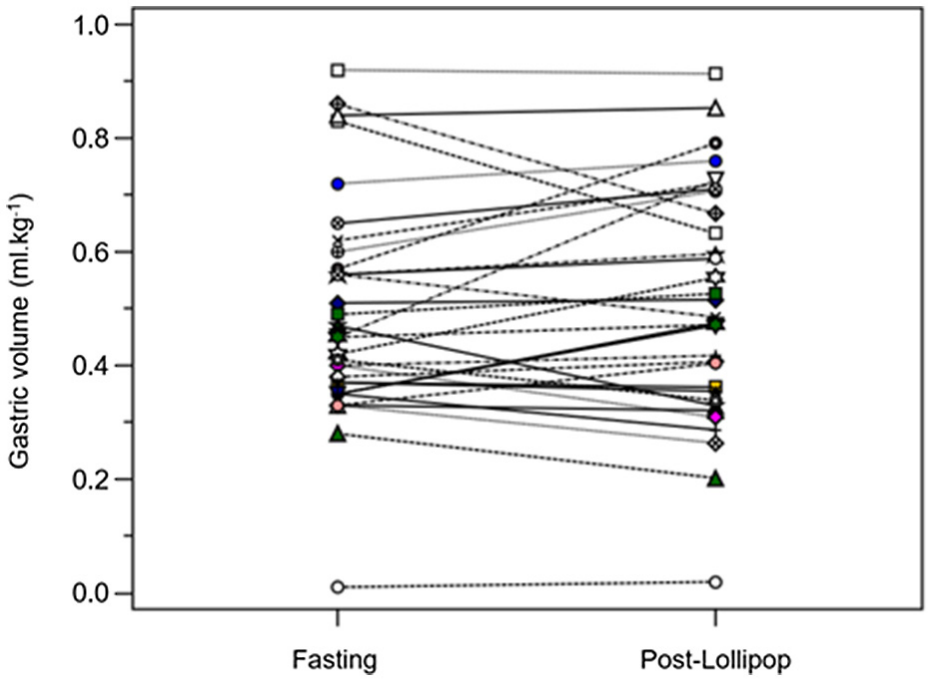
Dot and line diagram depicting change in antrum gastric volume in each subject.

**Figure 4:**
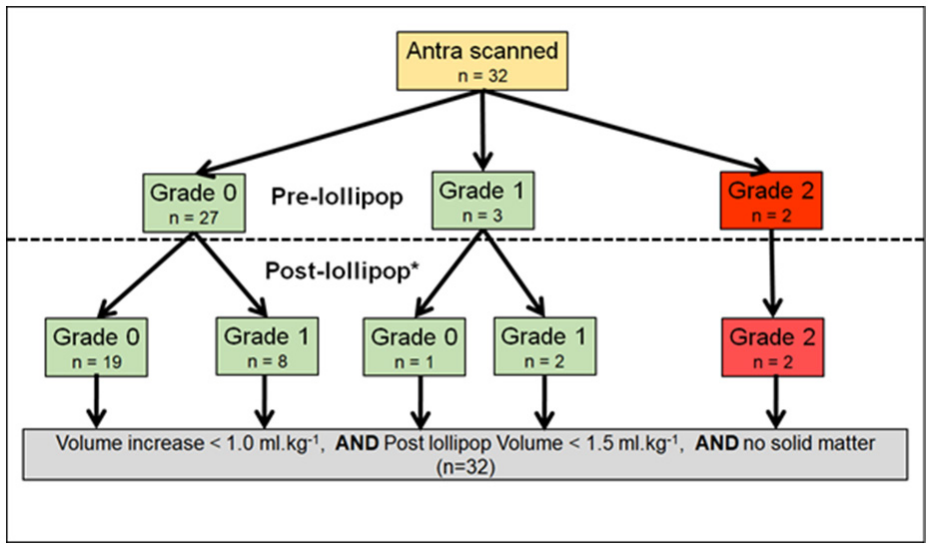
Flow diagram of the ultrasonic grading and volume measurements.

All 32 participants consumed their lollipops within 1 h, and all gastric antra were visualized successfully. No episodes of vomiting, regurgitation, or discomfort occurred during the study. Volume measurements are shown in Table 2. The gastric volume data were normally distributed. The mean volume change was 0.01 ml kg^−1^ (95% CI −0.02 to 0.05; *p* = .460) (Figure 2). Not one participant showed a volume change >1.0 ml kg^−1^ (range −0.20 to 0.28 ml kg^−1^) (Figure 3). The mean postlollipop volume was 0.51 ml kg^−1^ (95% CI 0.43 to 0.58) (Figure 4).

**Table 2:** Calculated gastric antrum volumes (ml kg^−1^ body weight), pre‐ and post‐lollipop

	Fasted (*n* = 32)	After lollipop (*n* = 32)
Mean (95% Confidence interval)	0.49 (0.42 to 0.56)	0.51 (0.43 to 0.58)
Standard deviation	0.19	0.21
Range	0.01 to 0.92	0.02 to 0.91
Mean difference (95% Confidence interval)	0.01 (−0.02 to 0.05)	
*p* (paired *t*‐test)	.460	

Twenty‐seven participants were classified as prelollipop Grade‐0, of whom 8 converted to Grade‐1 postlollipop. Three participants were prelollipop Grade‐1 of whom one converted to Grade‐0. The proportional change of prelollipop Grades 0 and 1 participants was statistically significant (McNemar test: *p* < .0001; 95% CI of the difference between proportions 49.8% to 83.5%). The status of the two participants who were prelollipop Grade‐2, did not change after the lollipop. Overall, the antral volumes of Grade‐1 participants were statistically significantly greater than those of Grade‐0 participants (Table 3). A summary of the grading and volume measurements are presented in Figure 3.

**Table 3:** Comparison of gastric volumes measured in Grade‐0, Grade‐1 and Grade‐2 gastric antra

	*n*	Median volume (ml kg^−1^)	Interquartile range (ml kg^−1^)	*p* (Kruskal–Wallis ANOVA)
Grade‐0	47	0.42	0.35 to 0.57	.0344^a^
Grade‐1	13	0.71	0.40 to 0.80	
Grade‐2	4	0.48	0.52 to 0.64	

^a^ Grade‐1 significantly different from Grade‐0 (Conover multiple comparisons).

## 4 DISCUSSION

We assessed participants' gastric volumes after an overnight fast, and 1 h after being offered a lollipop. To our knowledge, this is the first investigation regarding the effect of a carbohydrate lollipop on gastric volume in children, using ultrasound. We did not detect a significant change in gastric volumes. The volume changes met our a priori specification for gastric volume equivalence.

Gastric physiology is complex and differs between fasted and fed states.^21^ Saliva and gastric secretions, respectively, contribute up to 1 and 0.6 ml kg^−1^ h^−1^ of baseline volume**.** A GRV <1.5 ml kg^−1^ corresponds to baseline secretions and is regarded as low risk for aspiration.^11^, ^14^ A GRV >1.5 ml kg^−1^ corresponds to volumes exceeding fasted gastric volume and indicates high risk for aspiration.

According to the qualitative grading system, the two Grade‐2 participants should indicate high aspiration risks. However, not one participant experienced a postlollipop volume considered a risk for aspiration (increase >1 ml kg^−1^ or postlollipop volume >1.5 ml·kg^−1^).^11^, ^14^ Prior studies indicate that qualitative grading of gastric content is a useful preoperative screening tool, and that increases in qualitative antrum grading are accompanied by increased gastric volumes.^13^, ^14^, ^20^ ESA guidelines recommend qualitative grading for airway management decisions in patients with an unsure fasting status (weak recommendation, low‐quality evidence).^2^ However, measured volumes in Grade‐2 antra are often smaller than 1.5 ml kg^−1^, and prior studies recommend quantitative measurement should complement qualitative assessment in children.^13^, ^20^ Figure A1 in the Appendix S1 depicts our subjects' considerable overlap regarding volume measurements in the three grades. The two Grade‐2 participants were classified as Grade‐2 before and after consuming lollipops. Their volumes were small (Table 3 legend). Our few Grade‐2 numbers preclude statistical comparison with previous studies; however, those studies reveal considerable variation regarding Grade‐2 volumes (Table A3 in the Appendix S1).^13^, ^14^, ^20^ Our study supports the use of quantitative methods in addition to qualitative grading.^13^, ^20^

A randomized controlled trial compared control, placebo, and fentanyl lollipop effects on GRV and pH, using nasogastric tube aspiration.^22^ There was no significant increase in GRV between the fasted control and placebo carbohydrate lollipop groups. A statistically significant increased GRV was detected in the fentanyl lollipop group, that was clinically unimportant. There was no difference in gastric pH between the groups. Mean times to consume the lollipops were <20 min. Our study corroborates the negligible volume change after consumption of carbohydrate lollipops and the feasibility of children to completely consume them within an hour.

Providing preoperative carbohydrates to children, such as chewing gum and fluids, have been investigated. A meta‐analysis of 287 patients concluded that chewing gum increases GRV with statistical significance, but the increase is clinically unimportant. Gastric pH remains unchanged.^23^ Oral fluids have been investigated using ultrasound. Song and colleagues found a mean decrease of 0.24 cm^2^ in antral CSA (95% CI 0.06 to 0.43; *p* = .01) 2 h after an oral carbohydrate fluid regimen.^16^ Taye and colleagues found that after ingestion of fluid volumes between 3 and 5 ml kg^−1^, weight adjusted GRV returned to baseline after 50 min.^17^ Our lollipop volume was 3.5 ml, less than the volumes used in previous studies. Boiled sweets may increase saliva and gastric secretions during digestion.^21^ Our findings of no significant increase in gastric volume between prelollipop and postlollipop measurements, support other studies' findings of this increase in secretions not being clinically relevant, possibly due to gastric emptying promotion from carbohydrates.^16^, ^24^

Gastric antral ultrasound enables qualitative and quantitative assessment of gastric contents. It is reliable, reproducible, and noninvasive.^25^ It is used for preoperative assessment in both adults and children.^11^ We observed no significant change in gastric volume when using the CSA in the RLD position. Our mean gastric volumes prelollipop and postlollipop are similar to fasted means in studies that used magnetic resonance imaging^24^ and ultrasound.^13^, ^20^

Increased GRV is a contributing factor to aspiration risk, but it is evident that consuming a lollipop does not increase volumes above baseline values. Shortening fasting times for clear fluids may improve metabolism and hemodynamic tolerance to induction of anesthesia, while decreasing postoperative nausea and opioid use.^2^, ^4^, ^6^ Further research is required to establish whether a preoperative lollipop has similar advantages.

A possible weakness of our study is the lack of external validation of the equation used to calculate gastric volumes. It was derived during a study of 100 fasted children aged 11 months to 18 years, scheduled for upper gastro‐intestinal endoscopy, using sonographic antral CSA measurements.^14^ Gastric volumes were measured by endoscopic suction, regarded as the most accurate method. The coefficient of determination (*R* ^2^) was .6 in that study; however, their Bland–Altman analysis returned a bias of only 0.23 ml, and the differences between predicted and measured volumes were randomly scattered around the zero‐difference line. Within the range of our measurements (0.14–35 ml), only 2/87 (2.3%) of their measurement differences exceeded the limits of agreement (±22 ml). They demonstrated that RLD CSA resulted in the most successful visualization of the gastric antrum and that this measurement and age in months were the only significant predictors of aspirated gastric volume. Gender, height, weight, BMI, and supine CSA contributed little to the strength of the model and were removed during stepwise regression.^14^ An additional possible weakness is that the investigator was not blinded regarding pre‐ and postlollipop measurements, which may have introduced bias.

Strengths of our study include good quality data, due to ward staff supervision and the presence of the children's guardians, who made sure of their fasting status. The children were relaxed and cooperative, minimizing the role of anxiety on gastric emptying. Our participants ages ranged widely. By using participants as their own controls, our repeated measures design lessened potentially unknown confounding factors that can arise from individual differences between unmatched intervention and control groups. The “Spencer” equation^14^ was derived from an age group similar to our cohort.

Children in our study were healthy and scheduled for elective surgery. Emergency surgery per se is a risk factor for pulmonary aspiration, and our findings cannot be generalized to these cases. Our youngest participant was 2.4 years old; therefore, we cannot recommend administering lollipops to younger children, despite evidence that age does not influence stomach emptying.^26^ We used simple carbohydrate lollipops, thereby avoiding complex compounds such as fats, proteins, or gelatin that delay stomach emptying.

## 5 CONCLUSION

We conclude that children's gastric volumes do not increase after consuming a carbohydrate lollipop, nor does it result in increased risk of aspiration. Future research may investigate the effect of preoperative lollipops on patient, caretaker, and staff experience perioperatively, its possible metabolic benefits and the effect on post operative nausea and analgesic requirements.

## AUTHORS' CONTRIBUTIONS

P.O. and A.B. involved in study conception and study design. P.O. involved in data acquisition. P.O. and J.C. involved in data analysis and wrote the manuscript. P.O, A.B., and J.C. involved in final approval of published version.

## CONFLICT OF INTEREST

None to declare.

## Supporting information


Appendix S1

## Data Availability

The data that support the findings of this study are openly available in SUNScholar Research Repository at https://scholar.sun.ac.za, reference number http://hdl.handle.net/10019.1/125179.
