# Supplementary material for: Effect of a carbohydrate lollipop on the gastric volume of fasted pediatric patients
Source: Paediatr Anaesth. 2022 May 20;32(9):1031–7. doi: 10.1111/pan.14479 (PMC9545620; doi:10.1111/pan.14479)
Supplement: Supplementary file 1 — Appendix S1 [file PAN-32-1031-s001.docx]

# Appendices

1. Table A1: Statistical tests employed.

| **Data analysis** | **Test(s)** |
| --- | --- |
| Normal distribution of data | Shapiro-Wilk |
| Group equal variances | F-test |
| Gastric volumes, pre- and post-lollipop consumption. | 1. 95% Confidence interval of the differences between means 2. Paired t-test |
| Antral volumes (ml.kg^-1^) of participants categorised as Grade-0 to Grade-2 risk by ultrasound. | 1. Kruskal-Wallis one way analysis of variance 2. Conover post hoc multiple comparison (Reference: Conover WJ (1999) Practical nonparametric statistics, 3rd edition. New York: John Wiley & Sons |
| Proportion of Grade-0 participants whose ultrasound gradings increased. | 1. McNemar test for paired proportions. 2. 95% Confidence interval of the difference between proportions (Reference: Sheskin DJ (2011) Handbook of parametric and nonparametric statistical procedures. 5th ed. Boca Raton: Chapman & Hall) |

1. Table A2: Procedure types of participants

| **Procedure Type** | **Total (n=32)** |
| --- | --- |
| Circular Frame adjustment | 4 |
| Hardware Removal | 4 |
| Leg Traction | 7 |
| MRI of Limb | 6 |
| ORIF for non-union | 3 |
| Leg Ulcer Care | 4 |
| Epiphysiolysis | 1 |
| Nerve Exploration | 1 |
| Topical Eye Treatment | 1 |
| Hip SPICA | 1 |

1. Table A3: Previous studies grade-2 antrum volume estimation

| Study | N | Mean Grade-2 volume  (ml.kg^-1^) | 95% CI |
| --- | --- | --- | --- |
| Spencer et al. | 9 | 1.5 | 0.8 to 2.2 |
| Desgranges et al. | 9 | 0.44 | 0.33 to 0.55 |
| Bouvet et al. | 6 | 0.91 | 0.30 to 1.5 |

1.
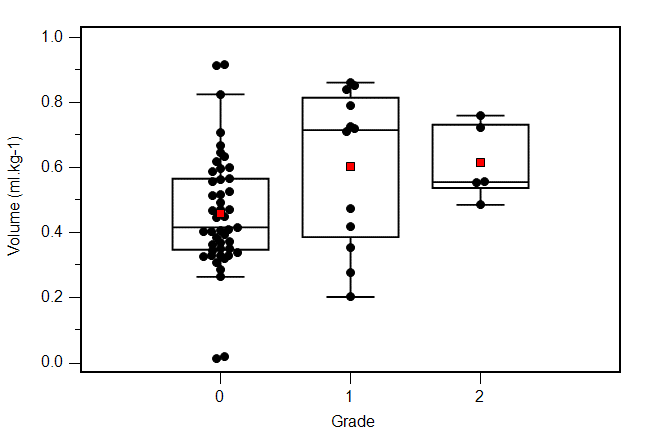
Figure A1 - Distributions of gastric volumes for the three grades.
